# Determinants of Organophosphorus Pesticide Urinary Metabolite Levels in Young Children Living in an Agricultural Community

**DOI:** 10.3390/ijerph8041061

**Published:** 2011-04-08

**Authors:** Asa Bradman, Rosemary Castorina, Dana Boyd Barr, Jonathan Chevrier, Martha E. Harnly, Ellen A. Eisen, Thomas E. McKone, Kim Harley, Nina Holland, Brenda Eskenazi

**Affiliations:** 1 Center for Environmental Research and Children’s Health (CERCH), School of Public Health, University of California at Berkeley, 1995 University Avenue, Berkeley, CA 94720, USA; E-Mails: rcastori@berkeley.edu (R.C.); chevrier@berkeley.edu (J.C.); eeisen@berkeley.edu (E.A.E.); kharley@berkeley.edu (K.H.); ninah@berkeley.edu (N.H.); eskenazi@berkeley.edu (B.E.); 2 Rollins School of Public Health, Emory University, 1518 Clifton Road, Atlanta, GA 30322, USA; E-Mail: dbbarr@emory.edu; 3 Environmental Health Investigations Branch, California Department of Public Health, 850 Marina Bay Parkway, Richmond, CA 94804, USA; E-Mail: martha.harnly@cdph.ca.gov; 4 Division of Environmental Health, School of Public Health, University of California at Berkeley, 50 University Hall, Berkeley, CA 94720, USA; 5 Department of Environmental Health, Harvard School of Public Health, 665 Huntington Ave, Boston, MA 02115, USA; 6 Lawrence Berkeley National Laboratory, 1 Cyclotron Road, Berkeley, CA 94720, USA; E-Mail: temckone@lbl.gov

**Keywords:** children, organophosphorus, pesticides, exposure, agriculture, biomarkers, diet

## Abstract

Organophosphorus (OP) pesticides are used in agriculture and several are registered for home use. As young children age they may experience different pesticide exposures due to varying diet, behavior, and other factors. We measured six OP dialkylphosphate (DAP) metabolites (three dimethyl alkylphosphates (DMAP) and three diethyl alkylphosphates (DEAP)) in urine samples collected from ∼400 children living in an agricultural community when they were 6, 12, and 24 months old. We examined bivariate associations between DAP metabolite levels and determinants such as age, diet, season, and parent occupation. To evaluate independent impacts, we then used generalized linear mixed multivariable models including interaction terms with age. The final models indicated that DMAP metabolite levels increased with age. DMAP levels were also positively associated with daily servings of produce at 6- and 24-months. Among the 6-month olds, DMAP metabolite levels were higher when samples were collected during the summer/spring *versus* the winter/fall months. Among the 12-month olds, DMAP and DEAP metabolites were higher when children lived ≤60 meters from an agricultural field. Among the 24-month-olds, DEAP metabolite levels were higher during the summer/spring months. Our findings suggest that there are multiple determinants of OP pesticide exposures, notably dietary intake and temporal and spatial proximity to agricultural use. The impact of these determinants varied by age and class of DAP metabolite.

## 1. Introduction

Public health concerns about pesticide exposure to young children have received increased attention following the publication of “Pesticides in the diets of infants and children” in 1993 [1]. In 1996, the U.S. Food Quality Protection Act (FQPA) required the U.S. Environmental Protection Agency (U.S. EPA) to set food tolerances that account for dietary and non-dietary exposure and protect sensitive populations [2]. Biomonitoring studies have confirmed that children are widely exposed to pesticides, including organophosphorus (OPs), pyrethroid, fungicide, and organochlorine pesticides [3–6]. Diet is an important source of pesticide exposure in children. For example, Lu *et al.* [7] reported that the median urinary concentrations of the specific metabolites for malathion and chlorpyrifos decreased to undetectable levels after the introduction of organic diets in school-aged children. Several studies have confirmed that children may also be exposed to pesticide contamination in home and daycare environments [8–13].

Children living in agricultural areas may also be exposed to pesticides through drift during applications or volatilization from nearby fields and parental take-home exposures [10,11,14–22]. Lu *et al.* [18] found that children (9 months to six years old) who live in agricultural communities had five times higher OP metabolite levels in their urine compared to children who resided in nonagricultural communities. These researchers also found higher residential OP pesticide contamination and/or elevated urinary metabolite levels in children living near orchards [18]. Higher exposure to children living in agricultural areas has raised environmental justice concerns and has resulted in proposals to define farmworker children as a vulnerable population that need additional protections by the U.S. EPA [23].

Identifying pesticide exposure determinants is needed to identify sources and pathways of pesticide exposure in children and contribute to policies aiming to reduce exposure. To date, no longitudinal studies have investigated factors associated with pesticide exposure in very young children. We hypothesize that exposure factors will vary over time given the changes in diet, behavior, and family practices that occur as children age [24]. In this study, we report levels of OP pesticide metabolites in 6, 12, and 24 month old children (n = 417) participating in the CHAMACOS birth cohort study in the Salinas Valley of California, an agricultural area. We examined potential determinants of exposure associated with OP urinary metabolite levels at each age point, including sex, child behavior, diet, home pesticide use, season, parental work status, and proximity of homes to fields. We focused on OPs because they are commonly used in the Salinas Valley and were the first pesticide class re-examined under the FQPA.

## 2. Methods

### 2.1. Participants and Recruitment

The Center for the Health Assessment of Mothers and Children of Salinas (CHAMACOS) is a longitudinal cohort study investigating environmental exposures and health of pregnant women and their children living in the Salinas Valley, Monterey County, California [25]. Between October 1999 and November 2000, 601 pregnant women were enrolled in the CHAMACOS birth cohort study, resulting in 538 live births. Eligible women were ≥18 years old, <20 weeks gestation, Spanish- or English-speaking, eligible for Medi-Cal, receiving prenatal care at local community clinics, and planning to deliver at the county hospital in Salinas, California. We collected urine samples from 97% (420 of 434) of children at 6 months, 92% (407 of 441) of children at 12 months, and 92% (382 of 414) of children at 24 months of age. Participating children turned six months of age between August 2000 and December 2001; 12 months of age between February 2001 and June 2002; and, 24 months of age between February 2002 and June 2003. Written informed consent was obtained from all participants and the study was approved by the Committee for the Protection of Human Subjects at the University of California, Berkeley.

### 2.2. Interviews and Home Assessments

Mothers were interviewed when the children were 6, 12, and 24 months old. Interviews were conducted in Spanish or English by bilingual interviewers. Information collected included demographics, household enumeration, occupational status, whether work clothes were worn into the home, home pesticide use, presence of pets, daily servings of child fruit and vegetable consumption based on a modified food frequency questionnaire, time spent in child care, location of child care relative to fields, and frequency of hand washing and how often child fingers, hands, or toes are placed in the mouth. The interview also included a Child Behavior Checklist (CBCL) which uses a standardized format to assess parent-reported behavioral characteristics of children. Based on the CBCL, we selected child temperament indicators that we hypothesized could be associated with behaviors that affect pesticide exposure: “Can’t sit still, restless, or hyperactive”, “Gets into everything”, “Quickly shifts from one activity to another”, and “Underactive, slow moving, or lacks energy.”

Shortly after each interview, study staff conducted a home inspection. Recorded information included distance between the home and agricultural fields, carpeting, housekeeping quality, and a detailed inventory of home pesticides [26]. Home visits were completed for 87%, 84%, and 87% of the enrolled children at 6-, 12-, and 24-months, respectively.

### 2.3. Meteorological Data

Based on previous analyses that showed inverse associations between rainfall and air concentrations of OP pesticides [27], we hypothesized that daily rainfall may be associated with lower urinary metabolite levels. We obtained daily rainfall amounts near each home from the California Climate Data Archive [28]. We also examined season (Spring/Summer *versus* Fall/Winter) as a potential determinant of exposure since most agricultural pesticide use in this region occurs in spring and summer [29].

### 2.4. Child Urine Sample Collection

Random spot urine samples were collected from each child at 6, 12, and 24 months of age. A standard infant urine collection bag (Hollister, Libertyville, IL) was used during the study visit. If the child could not provide the sample during the visit, a spot sample was collected on the next day at the child’s home. Upon collection, urine samples were aliquotted and stored at −80 °C until analysis.

### 2.5. Laboratory Analysis

Urine samples were analyzed by the Centers for Disease Control and Prevention in Atlanta, Georgia. We measured six non-specific DAP metabolites of OP pesticides (three dimethyl alkylphosphate (DMAP) metabolites: dimethylphosphate (DMP); dimethyl-dithiophosphate (DMDTP); dimethylthiophosphate (DMTP); and three diethyl alkylphosphate (DEAP) metabolites: diethylphosphate (DEP); diethyldithiophosphate (DEDTP); and diethyl-thiophosphate (DETP)) by isotope dilution gas chromatography-tandem mass spectrometry (GC-MS/MS) [30]. We measured DAPs, rather than pesticide-specific metabolites, because there are no laboratory methods to measure specific metabolites of several OP pesticides used in the study area, such as oxydemeton-methyl. Approximately 80% of the OP pesticides used in the Salinas Valley devolve to a DAP metabolite (Supplementary Material, Table S1). Creatinine concentrations were determined in urine using a commercially available diagnostic enzyme method (Vitros CREA slides, Ortho Clinical Diagnostics, Raritan, NJ).

Laboratory quality control included repeat analysis of three in-house urine pools enriched with known amounts of pesticide residues whose target values and confidence limits were previously determined. The validity of each analytical run was determined using the Westgard rules for quality control [31]. The limits of detection (LODs) ranged from 0.08 μg/L for DMDTP to 1.1 μg/L for DMTP. Metabolite levels below the LOD were randomly imputed based on a log-normal probability distribution. Because individual OP pesticides can devolve to more than one DAP metabolite, we summed the DAPs on a molar basis to reflect total DMAP or DEAP metabolites. Frozen field blanks, prepared earlier by CDC, were defrosted, re-packaged in the field in a manner identical to collection procedures for actual samples, and then shipped blinded to CDC. The mean levels of individual DAP metabolites in 57 blank field samples were <2 μg/L. The median values of the DAP metabolites in the field blanks were all below the detection limit.

### 2.6. Data Analysis

All data analyses were performed with Stata Version 10 (StataCorp LP, College Station, TX). We first computed descriptive statistics and percentiles for individual and total DMAP and DEAP metabolites at each sampling time point. We used Pearson correlations and ANOVA to assess bivariate associations between the metabolite levels (log_10_-transformed) and potential exposure determinants selected *a priori*, including sex, age, produce intake, breastfeeding, season, distance to agricultural fields, occupation of household members, wearing work clothes or shoes into the home, home pesticide use, presence of carpets, presence of pets, and housekeeping quality. We examined *post facto* additional determinants which may be related to drift of pesticides from fields, including daily rainfall, behaviors which may modify exposures (see Methods above), time spent in child care, and proximity of child care to agricultural fields [10,11,14–21,27].

We then constructed generalized linear mixed models (GLLAMM procedure in Stata Version 10 (StataCorp LP, College Station, TX) with log_10_-transformed DMAP or DEAP metabolite levels as the dependent variables and potential exposure determinants found to have significant (p < 0.1) bivariate relationships. The models included a random effects term to adjust for the lack of independence of repeated measures on the same subject. Because children’s development, diet, and behavior differ at different age points, we also examined whether age modified any associations, with 12-month olds (yes/no) and 24-month olds (yes/no) compared to 6-month olds as the reference. All interaction terms were included in the final DMAP and DEAP models. Based on the final models, we used linear combination equations to compute the percent differences in log DMAP and DEAP metabolites for the predictor variables to determine the effect of these predictors on metabolite levels among the 6-, 12- and 24-month old children. To assess bias due to loss to follow up, we ran the models with weights equal to the inverse probability of inclusion in the final sample at each time-point [32,33]. We then performed the analyses without the weights for comparison.

For statistical analyses, we present results that are not adjusted for creatinine. Analyses were repeated with creatinine-adjusted values to confirm our bivariate results. We also included urinary creatinine as an independent variable in the final multivariable mixed DMAP and DEAP models for comparison with models without the urinary creatinine variable [34].

## 3. Results

### 3.1. Demographic Characteristics

Table 1 presents the demographic characteristics of the CHAMACOS mothers (n = 460) when their children were 6-months old. Fifteen percent of mothers were employed as agricultural workers, and 66% percent shared a home with at least one agricultural worker. Sixty-six percent of mothers were living at or below the U.S. federal poverty threshold. The average ages (SD) of the children at the three sampling time points were 6.7 (1.1); 13.0 (1.7); and 24.6 (1.1) months, respectively.

### 3.2. Urinary Metabolite Concentration Data

Table 2 presents descriptive statistics for the total DEAP, DMAP and DAP molar concentrations at each sampling point. The DMAP metabolites were dominated by DMTP and DMP (68 and 50% detection frequency, respectively, at 6-months), whereas the DEAP metabolites were dominated by DEP (68% detection frequency at 6-months). Oxydemeton methyl, malathion, and dimethoate were the most common dimethyl OP pesticides used in the region, while chlorpyrifos and diazinon were the most common diethyls (Supplementary Material, Table S1). Most participants had at least one DAP detected with 6-, 12- and 24-month detection frequencies of 93%, 94% and 95%, respectively. Consistent with previous studies, the DMAP metabolite levels were higher than the DEAP metabolite levels [8,35,36].

DMAP metabolite levels were about three-fold higher at 24-months and two-fold higher at 12-months compared to the 6- month-old children. There is no clear pattern in the change in DEAP levels with age. Pearson correlations between the 6-, 12- and 24-month sampling time points ranged from −0.03 to 0.07 for the DEAP metabolites (p-values > 0.05), 0.07 to 0.22 for the DMAP metabolites, and 0.02 to 0.19 for the total DAP metabolites. These results suggest weak inter-sample correlations (Pearson r∼0.2) in DMAPs and total DAPs in urine collected at 12 and 24 months (p-values < 0.001). Within each sampling cross-section (6, 12, and 24 months), the DMAP and DEAP metabolites were moderately correlated: 0.29; 0.50; and 0.48 (all p < 0.001), respectively. The children’s 6- 12- and 24-month creatinine-adjusted urinary metabolite levels are presented in the Supplementary Material, Table S2.

### 3.3. Determinates of DMAP metabolites

The unadjusted relationships between covariates and DMAP and DEAP metabolite levels at the different ages are shown in the Supplementary Material, Tables S3 and S4; the only consistent association across the three ages was that DMAP metabolites were positively associated with the number of children’s daily servings of fruits and vegetables (fresh and processed combined) (Pearson r = 0.17, 0.12, and 0.12, respectively (p < 0.05)). For other exposure determinants, the relationship with DMAP metabolites varied with age category. At 6-month olds, DMAP metabolites were higher in females *versus* males (geometric mean = 22 *vs.* 15 nmol/L (p < 0.05)); in samples collected in spring/summer *vs.*winter/fall (25 *vs.* 13 nmol/L (p < 0.01)); when at least one agricultural worker lived in the home *vs.*none (21 *vs.* 11 nmol/L (p < 0.01)); when the mother currently worked as an agricultural worker *vs.* not (29 *vs.* 16 nmol/L (p < 0.01)); with the child having spent >15 hours/week in child care (25 *vs.* 16 nmol/L (p < 0.05)); and with having the child care facility ≤ 60 m from a field (45 *vs.* 17 nmol/L (p < 0.05)). DMAP metabolite levels were also associated with the number of household members wearing agricultural work clothes (r = 0.19, p < 0.01) and shoes inside the home and mean millimeters of rainfall in the week prior to urine collection (r = −0.16, p < 0.01). For 12-month olds, higher DMAP levels were found in those living < 60 meters *vs.* > 60 meters from an agricultural field (60 *vs.* 25 nmol/L (p < 0.05)). No other covariates including housekeeping quality, exposure-related behaviors, and temperament indicators as reported by the mother were associated with DMAP metabolite levels.

Table 3 (see also Supplementary Material, Figure S1) presents results from the multivariable GLLAMM analysis for DMAP levels. DMAP metabolites increased 59%, 36%, and 104% for each additional daily serving of fruits and vegetables among the 6-, 12- and 24-month old children; the results were not significant at 12-months. DMAP metabolites increased 56% when urine collection occurred during the spring/summer *versus* the winter/fall months (p-value < 0.05) for 6-month olds but not significantly at the other ages. For the 12-month old children, mean daily rainfall in the prior week was significantly associated with decreasing DMAP levels (−13% per millimeter of rain) and living ≤60 m *vs.* >60 m from an agricultural field was associated with increased levels (change = 192%; p-value < 0.01). No other covariates were related to DMAP levels at any of the ages. Overall, the DMAP multivariable model results were similar when creatinine was included as a covariate (results not shown).

### 3.4. Determinants of DEAP Metabolites

Supplementary Material, Table S4 presents the unadjusted relationships between covariates and DEAP levels at the different ages. At 6 months of age, there was a significant positive association between DEAP metabolites and having the child’s child care facility ≤60 m from an agricultural field (geometric mean = 18 *vs.* 8 nmol/L (p < 0.05)) and a negative association with rainfall measured locally during the period prior to urine collection. At 12 months of age, those living ≤60 m *vs.* >60 m from an agricultural field had significantly higher levels (26 *vs.*13 nmol/L (p < 0.05)). At 24 months of age, DEAP metabolites were significantly higher when urine samples were collected in the spring/summer *vs.*winter/fall (12 *vs.* 6 nmol/L (p < 0.01)) and were positively correlated with the number of daily servings of fruits and vegetables (Pearson r = 0.15 (p < 0.01)). No other covariates were associated at any of the ages including child temperament.

The multivariable DEAP model confirmed the univariate findings and were notably different than the DMAP findings (see Table 3 and Supplementary Material, Table S4 and Figure S2). Among the 6-month olds, mean daily rainfall (millimeters) was negatively associated with DEAP levels. Among the 12-month olds, similar to the DMAP findings, DEAP metabolite levels were 107% higher among 12-month olds living ≤60 m *vs.* >60 m from an agricultural field. Among the 24-month olds, DEAP metabolite levels were 109% higher in the spring/summer *versus* the winter/fall months, but, in contrast to the findings for the 6-month olds, DEAP metabolites was positively associated with average daily rainfall. No other significant associations were observed between children’s DEAP metabolites and predictors of exposure in multivariable models, including daily servings of fruits and vegetables (Table 3). Results were similar after creatinine adjustment (not shown).

## 4. Discussion

We investigated the relationship between potential exposure determinants and urinary (OP) pesticide metabolite levels in ∼400 children followed through infancy and toddlerhood living in an agricultural community. All children had detectable levels of OP metabolites in their urine. Consistent with previous studies, the DMAP metabolite levels were higher than the DEAP metabolite levels [8,35,36].

We observed three-fold higher DMAP levels in 24-month olds and two-fold higher levels in 12-month olds relative to 6 month olds; however DEAPs declined between 12 and 24 months. Nearby agricultural use of dimethyl and diethyl OP pesticides was generally stable over the study period, however, most residential uses of chlorpyrifos and diazinon, two diethyl OP pesticides, were cancelled [29,37–41]. CHAMACOS children turned 12 months during the first year of the residential ban, which was phased in gradually. Thus, the decrease in DEAP metabolite levels among 24-month olds may be related to reduced indoor contamination of chlorpyrifos and diazinon (both diethyl OP pesticides), due to the residential use ban. This hypothesis is supported by our finding in a separate study that chlorpyrifos and diazinon house dust levels declined in Salinas Valley homes between 2000 and 2006 [42]. However, the ontogenetic increase in DMAP levels cannot be explained by changes in dimethyl pesticide use which did not change substantially during this time. The increase in DMAP levels may be due to increasing exposure-related behaviors and changes in diet as the children age in an environment where dimethyl OP pesticide use was relatively constant.

Associations between the two classes of DAP metabolites (DEAPs and DMAPs) and exposure determinants were not consistent at different age points. Possible reasons include differences in usage patterns, physical-chemical properties of the pesticides, field degradation, environmental transport, and metabolism of the dimethyl *versus* the diethyl OP pesticides. For example, malathion, which devolves to a DMAP metabolite, has a relatively high vapor pressure compared to other OP pesticides, and, thus, may result in greater exposures via inhalation. The spring/summer season, when malathion use is higher, was associated with higher DMAP levels in six-month olds, who are not yet crawling, suggesting an inhalation exposure pathway. We also found that recent rainfall was associated with lower DMAP levels in the younger children, a finding consistent with our previous study that showed rainfall was associated with lower OP levels in air [27]. Together, these findings support the hypothesis that inhalation may be an important pesticide exposure route for very young children.

Overall, our findings suggest that agriculture-related determinants of pesticide exposure (e.g., proximity to field or occupational status) may be associated with measured exposure at some ages, but we did not observe consistent associations across age points, or between DMAP and DEAP metabolites. The high variability in pesticide application frequency and the nature of transient, non-persistent exposures in young children may create too much variability to statistically model the association of these variables and child exposures. In contrast, intake of fruits and vegetables was consistently and positively associated with both classes of urinary metabolites in children at all ages, and was statistically significant for DMAP metabolites in 6- and 24-month old children, suggesting that diet is an important pesticide exposure pathway. This finding is consistent with recent studies that indicate diet is an important source of pesticide exposure to children [7,43].

Few studies report levels of pesticide metabolites in children 6- to 24-months old. Median total DAP metabolite levels in the CHAMACOS children at 6, 12, and 24 months of age (36, 54, and 76 nmol/L, respectively) were lower than levels in 10 crawling infants and 10 toddlers sampled in the Salinas Valley in 2002 (130 and 100 nmol/L, respectively) [8]. These twenty children were from farmworker homes and sampled in the summer, when levels might have been higher; direct comparisons, however, are limited by the small sample size. Median total DMAP and DEAP metabolite levels in the CHAMACOS 6- to 24-month olds were lower by ∼30–70% than levels in children 24- to 72-months old living in Washington state agricultural or suburban areas [6,10,15]; however, the Washington children were older than the CHAMACOS participants and the samples were collected between 1997 and 1999, before restrictions on residential use of chlorpyrifos and diazinon were implemented. Thus, these populations may not be directly comparable. Creatinine-adjusted levels were similar to adjusted concentrations reported in 41 5- to 73-month old farmworker children living on the US/Mexico border [21]. Due to age differences, it was not possible to compare DAP levels in these CHAMACOS children with levels in older children studied by the National Health and Nutrition Examination Survey (NHANES) [5]. Representative pesticide-exposure studies of national and state-wide populations are needed to compare to regional or local studies in impacted communities.

Our study has several limitations. In a setting where multiple OP pesticides are used, measurement of the non-specific DAP metabolites does not provide information on exposure to the specific parent OP compound [44]. As noted above, the many OP pesticides used in the Salinas Valley have widely varying usage, environmental persistence, and physical-chemical properties [14,45], adding variability to biomonitoring measurements and possibly biasing statistical models toward null results. Future studies focusing on parent compounds or pesticide-specific metabolites may be able to clearly elucidate associations between individual pesticide use and exposure. Additionally, DAPs in urine may reflect exposure to preformed DAPs in the environment or food rather than exposure to the parent compound [43,46] and thus overestimate OP pesticide exposure. Finally, the modified food frequency questionnaire we used quantified maternal reported servings of fruits and vegetables consumed by the child each day, but was not calibrated to specific portion sizes. Thus, the use of reported servings in the analyses may have introduced uncontrolled variability. However, this type of non-differential exposure misclassification would tend to bias results toward the null hypothesis.

In conclusion, we found that children living in an agricultural area are likely exposed to OP pesticides from multiple pathways, and total urinary DAP, in particular DMAP, metabolite levels increased with age. Diet and regional pesticide use are possible exposure sources. Given the health benefits of fresh fruit and vegetable consumption, we do not suggest that children limit intake of these foods but encourage washing of all produce before eating. While the OP pesticide metabolite levels in this population do not appear significantly higher than other populations, there are limited reference data available to make valid comparisons. OP pesticide exposures in children have been associated with poorer neurodevelopmental outcomes [47–49]. Given the significance of these health studies, additional research is needed to better explain the trend of increasing OP urinary metabolites with age and the dietary, behavioral, and other factors that determine exposure.

## Figures and Tables

**Table S1.** OP pesticide usage in the Salinas Valley ^a,b,c^ and associated urinary dialkyl phosphate metabolites.

**Pesticide ^d^**	**Kilograms applied in 2001**	**% applied in 2001**	**Metabolites**
Azinphos-methyl	56	0.1	DMP, DMTP, DMDTP
Dimethoate	15,523	15.3	DMP, DMTP, DMDTP
Malathion	44,181	43.5	DMP, DMTP, DMDTP
Methidathion	6,449	6.3	DMP, DMTP, DMDTP
Methyl parathion	0	0	DMP, DMTP
Naled	7,749	7.6	DMP
Oxydemeton-methyl	26,244	25.8	DMP, DMTP
Phosmet	1,436	1.4	DMP, DMTP, DMDTP
Total dimethyls	101,638	100	
Chlorpyrifos	25,283	27.5	DEP, DETP
Diazinon	61,944	67.4	DEP, DETP
Disulfoton	4,634	5.1	DEP, DETP, DEDTP
Total diethyls	91,861	100	

a Includes agricultural, landscape maintenance, structural pest control and right-of-way pesticide usage (DPR 2001).

b Pesticide usage is reported in kilograms of active ingredient.

c Agricultural pesticide applications in California, representing 90% of all reported use, are geocoded to one-square-mile units based on the Public Land Survey System. Pesticide use is reported to the County Agricultural Commissioner’s office who then reports it to the California Department of Pesticide Registration (CDPR) of the California Environmental Protection Agency (EPA). To summarize agricultural pesticide use in the Salinas Valley, we identified the PLSS sections within the Salinas Valley, and abstracted from the California PUR dataset the pesticide use within the SV.

d OP pesticides that do not metabolize to dialkyl phosphate compounds (e.g., bensulide, acephate, *etc.*) are not listed.

**Table S2.** Children’s creatinine-adjusted urinary DAP metabolite levels at three time points, at 6, 12 and 24 months (nmol/g) ^a^.

	**Detection Frequency (%)**	**Geometric Mean**	**Percentiles**					
Child 6 months (N = 414)			Min	25	50	75	90	Max

DMP	49.9	<LOD	<LOD	<LOD	<LOD	124	407	7,289
DMTP	68.1	32.3	<LOD	<LOD	36.6	145	656	7,457
DMDTP	25.5	<LOD	<LOD	<LOD	<LOD	4.4	133	1,629
Total DMAP	79.1	92.6	<LOD	24.5	80.0	298	1,371	10,066
DEP	67.6	18.2	<LOD	<LOD	31.2	106	194	77,968
DETP	31.3	<LOD	<LOD	<LOD	<LOD	19.2	59.9	4,852
DEDTP	13.9	<LOD	<LOD	<LOD	<LOD	<LOD	16.8	3,552
Total DEAP	79.1	42.9	<LOD	14.2	59.3	139	297	78,010
Total DAP (N = 409)	93.3	200	<LOD	75.1	182	587	1,661	78,237

Child 12 months (N = 405)			Min	25	50	75	90	Max

DMP	62.7	37.3	<LOD	<LOD	42.8	163	459	9,559
DMTP	56.0	26.4	<LOD	<LOD	29.0	171	731	8,530
DMDTP	52.7	5.1	<LOD	<LOD	3.7	30.3	133	3,087
Total DM	76.8	114	<LOD	22.0	124	451	1,408	10,298
DEP	81.1	26.4	<LOD	13.2	40.3	90.0	167	1,597
DETP	73.1	10.4	<LOD	<LOD	15.4	32.7	65.7	641
DEDTP	33.6	2.1	<LOD	<LOD	<LOD	8.3	36.1	696
Total DE	91.4	61.7	<LOD	30.8	69.4	148	254	1,972
Total DAP (N = 404)	94.3	235	<LOD	77.9	228	638	1,744	10,552

Child 24 months (N = 381)			Min	25	50	75	90	Max

DMP	61.9	35.5	<LOD	<LOD	52.9	182	444	2,506
DMTP	92.1	72.5	<LOD	24.0	73.4	216	544	3,897
DMDTP	32.6	<LOD	<LOD	<LOD	<LOD	8.8	28.0	864
Total DMAP	94.5	150	<LOD	53.7	165.3	415	1,017	5,843
DEP	57.2	10.3	<LOD	<LOD	38.1	101	243	1,071
DETP	63.0	4.7	<LOD	<LOD	4.8	15.7	44.4	3,122
DEDTP	9.5	<LOD	<LOD	<LOD	<LOD	<LOD	4.3	494
Total DEAP	72.2	28.1	<LOD	<LOD	49.6	128	308	3,924
Total DAP (N = 381)	95.3	221	<LOD	95.5	230	550	1,371	5,944

a Detection limits from multiple batches of urinary metabolite data: DMP = 0.4–0.6 μg/L; DMTP = 0.2–0.3 μg/L; DMDTP = 0.08–0.1 μg/L; DEP = 0.1–0.2 μg/L; DETP = 0.1 μg/L; DEDTP = 0.1 μg/L.

**Table S3.** Summary of children’s geometric mean DMAP urinary metabolite levels (nmol/L) and exposure prediction factors ^a^.

	**6-Month**		**12-Month**		**24-Month**	
	**n (%)**	**GM (95th % CI) or Correlation^b^**	**n (%)**	**GM (95th % CI) or Correlation^b^**	**n (%)**	**GM (95th % CI) or Correlation^b^**
**Child’s sex**						
Boy	208 (50.2)	15.2 (12.0, 19.2)	198 (49.0)	30.1 (22.6, 40.3)	189 (49.6)	49.6 (39.0, 63.1)
Girl	206 (49.8)	21.5 (16.7, 27.7)*	206 (51.0)	23.3 (18.0, 30.1)	192 (50.4)	40.9 (33.0, 50.8)
**Daily servings of fruits and vegetables**						
Pearson r	414 (100)	0.17 **	404 (100)	0.12 *	381 (100)	0.12 *
**Breastfeeding at time of urine collection**						
No	231 (57.9)	16.9 (13.5, 21.1)	313 (78.6)	25.3 (20.3, 31.6)	367 (96.6)	45.0 (38.1, 53.0)
Yes	168 (42.1)	20.5 (15.4, 27.2)	85 (21.4)	30.9 (20.1, 47.5)	13 (3.4)	43.7 (18.9, 100.7)
**Season of urine collection**						
Winter / Fall	200 (48.3)	13.0 (10.1, 16.7) **	213 (52.9)	28.0 (21.5, 36.5)	179 (47.0)	45.4 (35.9, 57.3)
Spring / Summer	214 (51.7)	24.5 (19.4, 31.0)	190 (47.1)	25.0 (18.8, 33.2)	202 (53.0)	44.7 (35.8, 55.9)
**Distance between home and fields**						
>60 m (200 feet)	375 (90.8)	17.9 (14.9, 21.4)	370 (91.6)	24.5 (20.0, 30.0)	355 (94.2)	45.5 (38.5, 53.7)
≤60 m (200 feet)	38 (9.2)	21.4 (11.9, 38.7)	34 (8.4)	59.8 (34.3, 104.4) *	22 (5.8)	43.3 (20.1, 93.5)
**Agricultural worker living in the home**						
No	104 (25.2)	10.8 (7.7, 15.1)	99 (24.5)	27.4 (18.2, 41.2)	94 (24.8)	52.7 (37.8, 73.4)
Yes	308 (74.8)	21.4 (17.6, 26.1) **	305 (75.5)	26.1 (20.9, 32.5)	285 (75.2)	43.0 (35.7, 51.8)
**Mom currently works in agriculture**						
Other job or not working	339 (82.5)	16.3 (13.5, 19.6)	331 (83.8)	24.7 (20.0, 30.5)	293 (79.2)	50.6 (42.2, 60.7)
Works in agriculture	72 (17.5)	29.2 (18.6, 48.8) *	64 (16.2)	38.4 (22.2, 66.6)	77 (20.8)	30.7 (21.2, 44.4)
**Any fresh produce brought home from the field**						
No	324 (94.4)	20.1 (16.5, 24.4)	351 (89.1)	25.9 (21.0, 31.9)	310 (83.8)	49.0 (41.0, 58.6)
Yes	19 (5.6)	9.9 (4.5, 21.8)	43 (10.9)	32.4 (18.1, 58.0)	60 (16.2)	33.5 (22.6, 49.7)
**# farmworkers wearing work clothes/shoes inside**	408 (98.6)	r = 0.19 **	396 (98.0)	r = 0.04	329 (86.4)	r = −0.10
**Home OP pesticide use in the last six months**						
No	354 (98.3)	19.2 (15.9, 23.2)	341 (98.0)	26.5 (21.4, 32.8)	346 (99.4)	42.7 (36.2, 50.5)
Yes	6 (1.7)	20.9 (5.4, 80.8)	7 (2.0)	58.7 (11.1, 309.7)	2 (0.6)	--
**Carpet/rug in any room in house**						
No carpet or rug	31 (7.8)	25.2 (13.8, 45.8)	32 (7.9)	23.9 (12.3, 46.6)	44 (11.6)	52.4 (32.5, 84.4)
Either carpet or rug-not specified	366 (92.2)	17.9 (14.9, 21.6)	372 (92.1)	26.6 (21.8, 32.6)	336 (88.4)	43.9 (40.0, 52.2)
**Pets Any dog or cat currently in the home**						
No	404 (97.6)	17.9 (15.0, 21.4)	395 (97.8)	25.9 (21.4, 31.5)	371 (97.4)	45.6 (38.8, 56.7)
Yes	10 (2.4)	24.4 (6.8, 87.5)	9 (2.2)	58.2 (9.0, 375.3)	10 (2.6)	27.1 (7.6, 103.7)
**Average daily rainfall^c^**	391 (94.4)	r = −0.16 **	381 (94.3)	r = −0.08	368 (96.6)	r = 0.08
**Quality of housekeeping**						
Less clean/Average	261 (69.6)	20.7 (16.6, 25.9)	271 (74.2)	26.5 (20.9, 33.7)	165 45.8)	47.9 (38.0, 60.3)
More clean	114 (30.4)	14.7 (10.8, 20.1)	94 (26.8)	26.9 (17.8, 40.8)	195 54.2)	39.6 (31.3, 49.9)
**Child spends >15 hrs/wk in child care**						
No	294 (71.5)	15.8 (13.0, 19.2)	283 (71.5)	26.1 (20.8, 32.8)	204 (55.0)	48.1 (38.2, 60.5)
Yes	117 (28.5)	25.3 (17.7, 36.1) *	113 (28.5)	27.3 (18.5, 40.4)	167 (45.0)	41.8 (33.2, 52.6)
**Distance between child care and ag field**						
>60 m	389 (94.6)	17.2 (14.4, 20.4)	377 (95.2)	26.1 (21.3, 31.9)	339 (91.4)	45.2 (38.1, 53.6)
≤60 m	22 (5.4)	44.6 (18.6, 106.9) *	19 (4.8)	35.4 (17.5, 71.5)	32 (8.6)	44.6 (24.8, 79.9)
**How often child mouths fingers and toes**						
Almost never/some of the time	231 (56.1)	21.4 (16.8, 27.4)	286 (72.2)	27.2 (21.5, 34.3)	339 (91.1)	43.9 (37.0, 52.2)
Most of the time	181 (43.9)	14.3 (11.3, 18.1)	110 (27.8)	24.7 (17.1, 35.6)	33 (8.9)	64.0 (37.8, 108.4)

* p-value < 0.05.

** p-value < 0.01.

a P-values are from Pearson correlations or ANOVA of log transformed DMAP metabolite levels.

b Geometric means and 95^th^ confidence interval except when Pearson coefficient (r) is presented.

c Mean daily millimeters of rainfall measured in Salinas, CA during the 1 week period prior to urine collection.

**Table S4.** Summary of children’s geometric mean DEAP urinary metabolite levels (nmol/L) and exposure prediction factors ^a^.

	**6-Month**		**12-Month**		**24-Month**	
	**n (%)**	**GM (95th CI) or Correlation^b^**	**n (%)**	**GM (95th CI) or Correlation^b^**	**n (%)**	**GM (95th CI) or Correlation^b^**
**Child’s sex**						
Boy	205 (50.4)	8.3 (6.6, 10.4)	197 (48.9)	15.0 (12.3, 18.3)	189 (49.5)	9.8 (7.3, 13.0)
Girl	202 (49.6)	8.3 (6.6, 10.6)	206 (51.1)	13.5 (11.3, 16.1)	192 (50.4)	7.2 (5.5, 9.6)
**Daily servings of fruits and vegetables**						
Pearson r	407 (100)	0.07	403 (100)	–0.004	381 (100)	0.15 **
**Breastfeeding at time of urine collection**						
No	228 (58.2)	7.5 (5.9, 9.5)	314 (79.1)	13.6 (11.7, 15.8)	367 (96.6)	8.6 (7.1, 10.6)
Yes	164 (41.8)	10.0 (8.0, 12.5)	83 (20.9)	17.1 (13.0, 22.7)	13 (3.4)	3.1 (0.8, 12.4)
**Season of urine collection**						
Winter/Fall	198 (48.6)	7.3 (5.7, 9.3)	213 (53.0)	15.4 (13.0, 18.1)	179 (47.0)	5.8 (4.2, 7.8)
Spring / Summer	209 (51.4)	9.4 (7.5, 11.6)	189 (47.0)	13.2 (10.7, 16.3)	202 (53.0)	11.7 (9.1, 15.1) **
**Distance between home and fields**						
>60 m (200 feet)	368 (90.6)	8.8 (7.4, 10.4)	369 (91.6)	13.4 (11.7, 15.4)	355 (94.2)	8.4 (6.9, 10.4)
≤60 m (200 feet)	38 (9.4)	5.2 (3.2, 8.5)	34 (8.4)	26.4 (17.6, 39.6) **	22 (5.8)	7.2 (3.0, 17.3)
**Agricultural worker currently living in the home**						
No	104 (25.7)	7.9 (5.7, 10.9)	99 (24.6)	13.2 (9.8, 17.8)	94 (24.8)	9.8 (6.4, 15.0)
Yes	301 (74.3)	8.5 (7.0, 10.3)	304 (75.4)	14.5 (12.5, 16.8)	285 (75.2)	8.0 (6.4, 10.1)
**Mom's current work categories**						
Not working or other job	335 (82.9)	8.0 (6.7, 9.6)	331 (84.0)	14.0 (12.1, 16.3)	293 (79.2)	9.2 (7.3, 11.5)
Agricultural work	69 (17.1)	9.7 (6.6, 14.3)	63 (16.0)	15.3 (11.5, 20.4)	77 (20.8)	6.5 (4.2, 10.2)
**Any fresh produce brought home from the field**						
No	316 (94.3)	8.7 (7.2, 10.5)	350 (89.1)	14.1 (12.2, 16.3)	310 (83.8)	8.6 (6.9, 10.8)
Yes	19 (5.7)	4.8 (2.1, 10.9)	43 (10.9)	15.3 (10.3, 22.7)	60 (16.2)	8.6 (5.3, 13.9)
**# farmworkers wearing work clothes/shoes inside**	401 (98.5)	r = −0.01	395 (98.0)	r = −0.07	329 (86.4)	r = −0.001
**Home OP pesticide use in the last six months**						
No	348 (98.3)	8.8 (7.4, 10.5)	341 (98.0)	14.6 (12.6, 16.9)	346 (99.4)	8.1 (6.6, 10.0)
Yes	6 (1.7)	22.3 (6.3, 78.6)	7 (2.0)	13.9 (3.7, 52.1)	2 (0.6)	--
**Carpet/rug in any room in house**						
No carpet or rug	30 (7.7)	9.8 (4.8, 20.3)	33 (8.2)	14.1 (8.3, 24.0)	44 (11.6)	13.8 (7.7, 24.6)
Either carpet or rug-not specified	360 (92.3)	8.4 (7.1, 9.9)	370 (91.8)	14.2 (12.4, 16.3)	336 (88.4)	7.8 (6.3, 9.7)
**Pets - Any dog or cat currently in home**						
No	397 (97.5)	8.3 (7.0, 9.8)	394 (97.8)	14.0 (12.2, 16.0)	371 (97.4)	8.4 (6.8, 10.2)
Yes	10 (2.5)	8.0 (2.3, 27.8)	9 (2.2)	27.3 (12.8, 58.5)	10 (2.6)	10.1 (2.1, 47.3)
**Average daily rainfall^c^**	384 (94.3)	r = 0.15*	380 (94.3)	r = −0.05	368 (96.6)	r = 0.07
**Quality of housekeeping**						
Less clean/Average	256 (69.4)	8.6 (6.9, 10.5)	271 (74.5)	13.7 (11.5, 16.1)	165 (45.8)	10.1 (7.6, 13.5)
More clean	113 (30.6)	9.8 (7.2, 13.3)	93 (25.6)	17.2 (13.4, 22.0)	195 (54.2)	7.0 (5.2, 9.3)
**Child spends >15 hrs/wk in child care**						
No	289 (71.5)	8.1 (6.7, 9.8)	283 (71.7)	13.8 (11.8, 16.1)	204 (55.0)	8.8 (6.7, 11.6)
Yes	115 (28.5)	8.8 (6.4, 12.1)	112 (28.4)	15.2 (11.7, 19.8)	167 (45.0)	8.2 (6.0, 11.1)
**Distance between child care and ag field**						
>60 m	382 (94.6)	8.2 (6.9, 9.7)	376 (95.2)	14.1 (12.3, 16.1)	339 (91.4)	8.8 (7.1, 10.8)
≤60 m	22 (5.5)	17.7 (8.7, 36.0) *	19 (4.8)	16.5 (9.4, 29.0)	32 (8.6)	6.3 (3.0, 13.3)
**How often child mouths fingers and toes**						
Almost never/some of the time	228 (56.3)	8.5 (6.8, 10.6)	286 (72.4)	14.8 (12.7, 17.3)	339 (91.1)	8.0 (6.5, 9.9)
Most of the time	177 (43.7)	7.9 (6.2, 10.1)	109 (27.6)	12.6 (9.7, 16.5)	33 (8.9)	15.3 (7.1, 32.8)

* p-value < 0.05.

** p-value < 0.01.

a P-values are from Pearson correlations or ANOVA of log transformed DEAP metabolite levels.

b Geometric means and 95^th^ confidence interval except when Pearson coefficient (r) is presented.

c Mean daily millimeters of rainfall measured in Salinas, CA during the 1 week period prior to urine collection.

**Table 1.** Demographic characteristics of participating families (n = 460).

**Characteristic**	**n**	**(%)**
Mother’s age (mean (SD) = 26.5 (5.2) years) ^a^		

18–24	207	(45.0)
25–29	142	(30.9)
30–35	83	(18.0)
35+	28	(6.1)

Mother’s country of birth		

Mexico	392	(85.2)
United States	60	(13.0)
Other	8	(1.7)

Mother’s length of residence in the United States ^b^		

Less than 5 years	214	(46.5)
5 or more years	246	(53.5)

Mother’s highest level of education ^b^		

Some elementary school (grades 1 to 6)	207	(45.0)
Grades 7 to 12	163	(35.4)
High school graduate	90	(19.6)

Language spoken at home		

Spanish mostly	409	(88.9)
English mostly	25	(5.4)
Both equally	21	(4.6)
Other	5	(1.1)

Family income relative to federal poverty threshold ^c^		

At or below poverty threshold	302	(65.6)
Above poverty threshold but below 200% of poverty	114	(24.8)
200% poverty threshold or greater	4	(0.9)
Not reported	40	(8.7)

Total number of household members when child is 6-months old		

2–3	30	(6.5)
4–5	129	(28.0)
6–9	205	(44.6)
10 or more	61	(13.3)
Not reported	35	(7.6)

Mother’s work status when child is 6-months old		

Not working	294	(63.9)
Field work	51	(11.1)
Ag work	18	(3.9)
Other work	64	(13.9)
Not reported	33	(7.2)

Other agricultural workers in household when child is 6-months old		

0	124	(27.0)
1–3	246	(53.5)
4–9	57	(12.4)
10 or more	1	(0.2)
Not reported	32	(7.0)

a At time of child’s birth.

b During pregnancy (*i.e.*, at time of entry into the CHAMACOS project).

c Poverty thresholds were calculated using the U.S. Department of Health and Human Services’ (HHS) level for the year 2000.

**Table 2.** Children’s urinary DAP metabolite levels at three time points, at 6, 12 and 24 months (nmol/L) ^a^.

	**Detection Frequency (%)**	**Geometric Mean**	**Percentiles**				
Child 6 months (N = 416)			25	50	75	90	Max

DMP	49.9	<LOD	<LOD	<LOD	26.8	97.1	1,291
DMTP	68.1	6.4	<LOD	6.8	27.0	153	4,059
DMDTP	25.5	<LOD	<LOD	<LOD	0.6	22.4	777
Total DMAP	79.1	18.5	5.4	15.2	62.0	293	5,329
DEP	67.6	3.6	<LOD	6.5	17.2	43.7	17,777
DETP	31.3	<LOD	<LOD	<LOD	5.4	10.8	311
DEDTP	13.9	<LOD	<LOD	<LOD	<LOD	3.6	1,041
Total DEAP	79.1	8.6	<LOD	10.8	25.7	58.8	17,786
Total DAP (N = 409)	93.3	40.0	13.7	36.1	110	356	17,838

Child 12 months (N = 404)			25	50	75	90	Max

DMP	62.7	8.7	<LOD	11.1	39.7	110	1,464
DMTP	56.0	6.1	<LOD	6.9	46.6	203	2,834
DMDTP	52.7	1.2	<LOD	1.0	7.6	34.5	463
Total DM	76.8	26.4	3.6	28.2	116	354	3,257
DEP	81.1	6.1	2.6	8.7	21.3	49.7	341
DETP	73.1	2.4	<LOD	4.3	6.9	14.6	110
DEDTP	33.6	<LOD	<LOD	<LOD	2.4	5.8	131
Total DE	91.4	14.2	7.7	16.2	32.9	67.7	389
Total DAP (N = 402)	94.3	54.3	19.2	53.9	160	433	3,298

Child 24 months (N = 381)			25	50	75	90	Max

DMP	61.9	10.7	<LOD	16.1	64.7	129	964
DMTP	92.1	21.8	7.5	20.6	67.2	178	1,656
DMDTP	32.6	<LOD	<LOD	<LOD	3.4	10.5	270
Total DMAP	94.5	45.0	13.2	50.3	141	309	2,305
DEP	57.2	3.1	<LOD	7.6	35.9	79.1	608
DETP	63.0	1.4	0.3	1.8	4.6	13.2	350
DEDTP	9.5	<LOD	<LOD	<LOD	<LOD	<LOD	44.4
Total DEAP	72.2	8.4	<LOD	12.2	45.6	92.1	724
Total DAP (N = 381)	95.3	66.3	23.1	75.8	192	391	2,380

a Detection limits from multiple batches of urinary metabolite data: DMP=0.4–0.6 μg/L; DMTP = 0.2–0.3 μg/L; DMDTP = 0.08–0.1 μg/L; DEP = 0.1–0.2 μg/L; DETP = 0.1 μg/L; DEDTP = 0.1 μg/L.

**Table 3.** Results from generalized linear mixed models of exposure predictors and DMAP and DEAP metabolites (log10) in children 6, 12- and 24-months old (416 children, 1201 urine samples).

	**6-Month %^a^ (95% CI)**	**12-Month %^a^ (95% CI)**	**24-Month %^a^ (95% CI)**
**DMAP metabolites**			

Daily servings of fruits and vegetables (≤ *vs.* > 1)	59.1* (9.6, 131.0)	35.8 (−24.3, 143.5)	104.0* (3.4, 302.2)
Agricultural worker living in the home (yes/no)	30.5 (−19.7, 111.9)	–5.7 (−42.0, 53.5)	–3.9 (−41.1, 57.0)
Average daily rainfall^b^	–8.7 (−16.9, 0.2)	–12.7* (−23.6, −0.3)	22.3† (−1.0, 51.0)
Child’s sex (Girl = 1)	32.3 (−6.9, 88.2)	–19.7†(−43.7, 14.7)	–20.2 (−46.0, 18.0)
Distance between home and fields (≤60 m = 1)	–16.7 (−54.7, 53.1)	192.3**† (53.4, 456.8)	–20.3 (−66.0, 87.1)
Farmworkers wearing work clothes / shoes inside^c^	11.7 (−0.7, 25.6)	4.6 (−6.4, 16.9)	–9.3† (−21.9, 5.3)
Spring/summer (*vs.* winter/fall)	56.1* (9.2, 123.2)	–12.0† (−38.9, 26.8)	4.0 (−29.7, 53.8)
Child care>15 hours/wk (yes/no)	7.8 (−29.5, 64.8)	4.6 (−31.4, 59.5)	–21.7 (−48.4, 18.8)
Child care distance to fields (≤60 m = 1)	69.9 (−27.3, 296.8)	2.6 (−57.6, 148.3)	9.1 (−47.6, 127.4)

**DEAP metabolites**			

Daily servings of fruits and vegetables (≤1 *vs.* >1)	25.0 (−12.0, 77.6)	3.1 (−40.5, 78.5)	56.5 (−17.3, 196.0)
Agricultural worker living in the home (yes/no)	–3.1 (−38.7, 53.0)	44.4 (−8.6, 128.2)	–12.9 (−45.0, 38.0)
Average daily rainfall^b^	–12.1** (−19.5, −4.0)	–8.2 (−19.0, 4.1)	25.6*† (3.0, 53.1)
Child’s sex (Girl = 1)	–6.5 (−32.9, 30.2)	–14.2 (−38.5, 19.6)	–22.0 (−45.9, 12.4)
Distance between home and fields (≤60 m = 1)	–41.4 (−66.8, 3.7)	106.9*† (13.2, 278.2)	–15.3 (−61.9, 88.2)
Farmworkers wearing work clothes / shoes inside^c^	–0.8 (−11.3, 10.9)	–10.6* (−19.4, −0.8)	5.0 (−8.7, 20.9)
Spring/summer (*vs.*winter/fall)	13.8 (−18.7, 59.5)	–20.3 (−43.4, 12.3)	109.0**† (45.0, 201.2)
Child care >15 hours/wk (yes/no)	–7.1 (−37.8, 38.9)	11.9 (−24.8, 66.6)	–19.7 (−45.7, 18.6)
Child care distance to fields (≤60m=1)	107.5 (−6.6, 361.1)	–2.9 (−57.6, 122.5)	–30.1† (−64.9, 39.2)

* p-value < 0.05;

** p-value < 0.01

† Significant interaction with age (p < 0.1) based on two indicator variables created to examine whether age modified any associations: 12-months old (yes/no) and 24-month olds (yes/no) compared to 6-month olds as the reference.

a Percent change in DMAP or DEAP metabolite levels associated with 1-unit increase or a yes/no difference in exposure characteristic.

b Mean daily millimeters of rainfall measured in Salinas, CA during the 1 week period prior to urine collection.

c Number of farmworkers in household who wear agricultural work clothes or shoes inside the home.

## Notes Added in Proof

The maximum value for the 6 month DEP, DEAP, and DAP is driven by a high value (Table 2 and Supplementary Material, Table S2). We have no reason to believe the measurement was incorrect.

## Supplementary Material

Figure S1. Percent change (and 95% CI) in DMAP metabolite per unit change or yes/no difference in predictor variable by child’s age. * p-value < 0.05.

Figure S2. Percent change (and 95% CI) in DEAP metabolite per unit change or yes/no difference in predictor variable by child’s age. * p-value < 0.05.
