# Supplementary figures and images for: Determinants of Organophosphorus Pesticide Urinary Metabolite Levels in Young Children Living in an Agricultural Community
Source: Int J Environ Res Public Health. 2011 Apr 8;8(4):1061–83. doi: 10.3390/ijerph8041061 (PMC3118878; doi:10.3390/ijerph8041061)

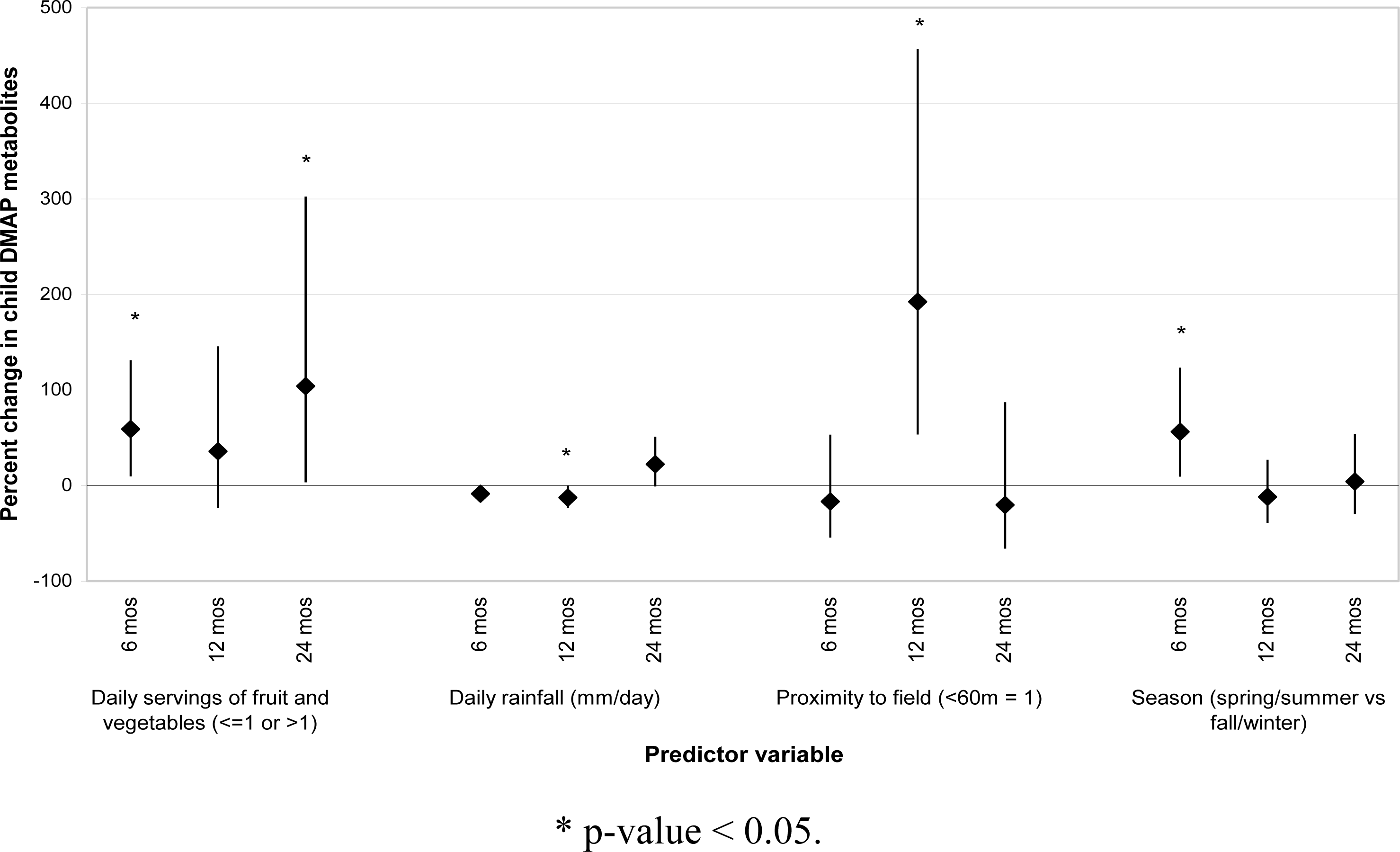

Supplement: Figure S1. — Percent change (and 95% CI) in DMAP metabolite per unit change or yes/no difference in predictor variable by child’s age. * p-value < 0.05. [file ijerph-08-01061s001.tif]

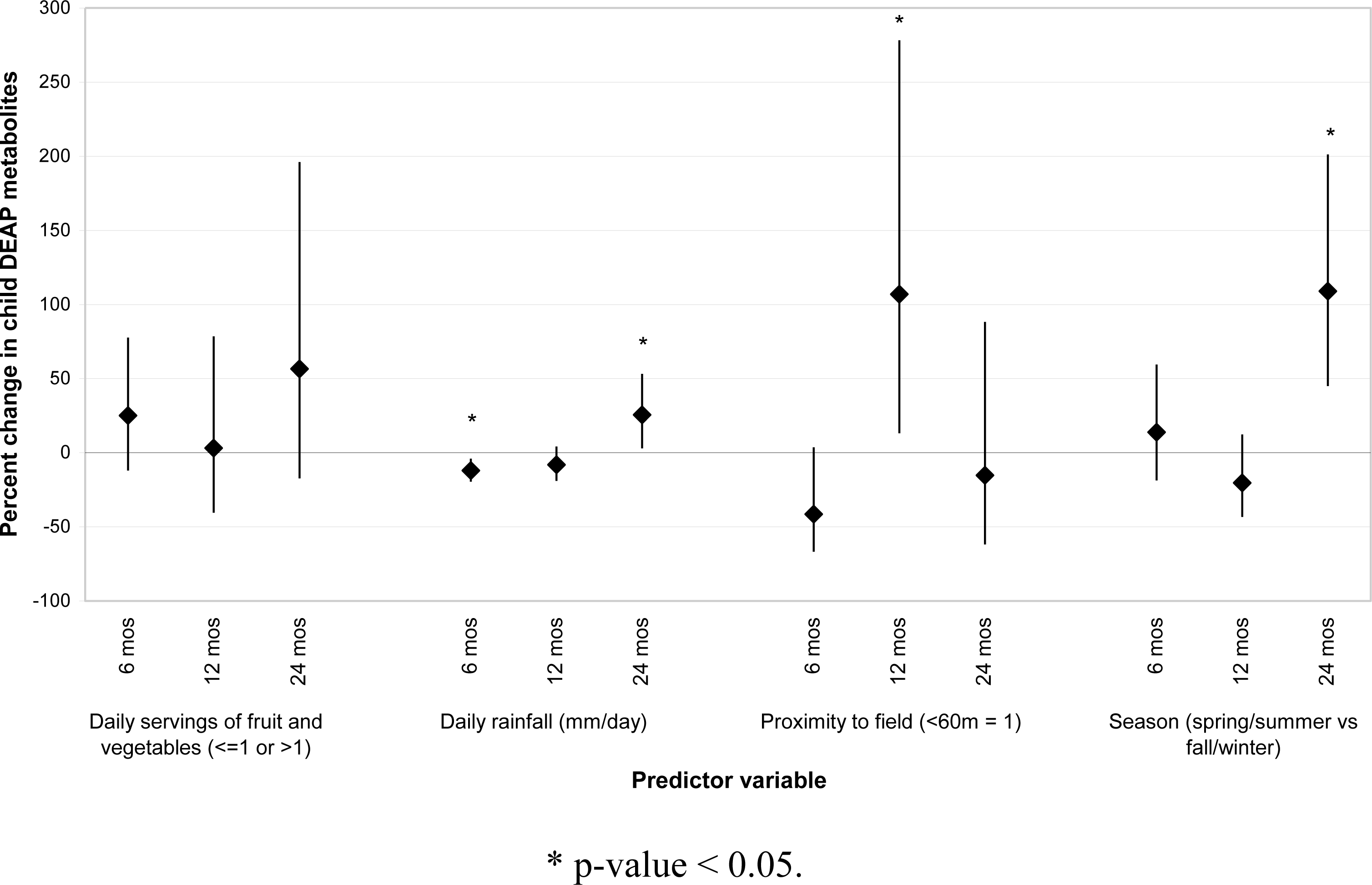

Supplement: Figure S2. — Percent change (and 95% CI) in DEAP metabolite per unit change or yes/no difference in predictor variable by child’s age. * p-value < 0.05. [file ijerph-08-01061s002.tif]
